# Concentration-dependent effects of narciclasine on cell cycle progression in *Arabidopsis *root tips

**DOI:** 10.1186/1471-2229-11-184

**Published:** 2011-12-28

**Authors:** Xiaofan Na, Yanfeng Hu, Kun Yue, Hongxia Lu, Pengfei Jia, Huahua Wang, Xiaomin Wang, Yurong Bi

**Affiliations:** 1 School of Life Sciences, Lanzhou University, Lanzhou 730000, People's Republic of China

## Abstract

**Background:**

Narciclasine (NCS) is an Amaryllidaceae alkaloid isolated from *Narcissus tazetta *bulbs. NCS has inhibitory effects on a broad range of biological activities and thus has various potential practical applications. Here we examine how NCS represses plant root growth.

**Results:**

Results showed that the inhibition of NCS on cell division in *Arabidopsis *root tips and its effects on cell differentiation are concentration-dependent; at low concentrations (0.5 and 1.0 μM) NCS preferentially targets mitotic cell cycle specific/cyclin complexes, whereas at high concentration (5.0 μM) the NCS-stimulated accumulation of Kip-related proteins (KRP1 and RP2) affects the CDK complexes with a role at both G1/S and G2/M phases.

**Conclusions:**

Our findings suggest that NCS modulates the coordination between cell division and differentiation in *Arabidopsis *root tips and hence affects the postembryonic development of *Arabidopsis *seedlings.

## Background

The growth and development of multicellular organisms depends on the spatiotemporal coordination of cell proliferation, cell differentiation, and subsequent cell specialization [1]. During plant postembryonic development, meristematic tissues provide new cells for growth at both ends of the main body axis. Stem cells in the root meristem generate transit-amplifying cells, which undergo additional divisions in the proximal meristem, and differentiate in the meristem transition zone that encompasses the boundary between dividing and expanding cells in different cell files. The balances between cell proliferation, cell cycle arrest and differentiation to maintain the organogenetic program depend on the coordination of gene expression, posttranslational modification, and specific proteolysis of cell cycle regulators. The retinoblastoma (RB)-E2F pathway is one of the most important regulatory pathways that control and couple cell division and cell differentiation [2,3]. The E2F and DP proteins interact to form active transcription factors that bind to various gene promoters and regulate the expression of genes required for cell cycle progression. The RB protein binds to E2F proteins, masking the transactivation region and blocking the transcriptional activity. This repression can be released by phosphorylation of the RB protein, catalyzed by cyclin-dependent kinases (CDKs), and formation of a functional E2F-DP heterodimer [2,4-8].

The CDK activity can be modulated by several mechanisms, including phosphorylation, cyclin degradation, or association with CDK inhibitory proteins [9-12]. CDK inhibitory proteins have also been proven to be important regulators of the endo-reduplication cycle in several organisms. Proteins related to the class of mammalian Kip/Cip CDK inhibitors have been identified in plants and designated Kip-related proteins (KRPs) [13,14]. Despite the low sequence homology with their mammalian counterparts, KRPs are *bona fide *functional orthologs of the Kip/Cip proteins that are capable of inhibiting the CDK activity both in vitro and in vivo [13,15-18].

Amarallidacae alkaloids are widely present in the plant kingdom and have important biological properties such as acetylcholinesterase inhibitory activity, cytotoxicity, antitumoral activity and so on [19]. Narciclasine (NCS) is an Amaryllidaceae alkaloid isolated from *Narcissus tazetta *bulbs and also exists in the genera *Galanthus*, *Haemanthus*, *Leucojum*, *Pancratium*, *Sprekelia*, *Sternbergia *and *Vallota *[20]. Previous studies showed that NCS possesses antimitotic [21] and antiviral functions [22], inhibits protein synthesis in rabbit reticulocyte and yeast cell-free systems [23], induces apoptosis-mediated cytotoxicity in certain human cancer cells [24] and activates Rho and stress fibers in glioblastoma cells [25]. McLachlan et al. showed that pancratistatin, of which the chemical structure is very close to NCS, induces rapid apoptosis in SHSY-5Y neuroblastoma cells at pharmacologic doses [26]. These pervious researches mainly focus on the anticancer potential of NCS, but show little interest in its function in plants. More recently, a broad range of inhibitory effects of NCS in plant were found, including inhibition of seed germination and seedling growth in rice and Chinese cabbage [27]; and accumulation of chlorophylls and chloroplast proteins in wheat [28]. However, little is known about the mechanism of NCS action in plant cells. In the present study, we provide evidence that NCS inhibits post-embryonic development by affecting the balance between cell division and differentiation in *Arabidopsis *root tips.

## Results

### Effects of NCS on postembryonic development of *Arabidopsis *roots

**Figure 1:**
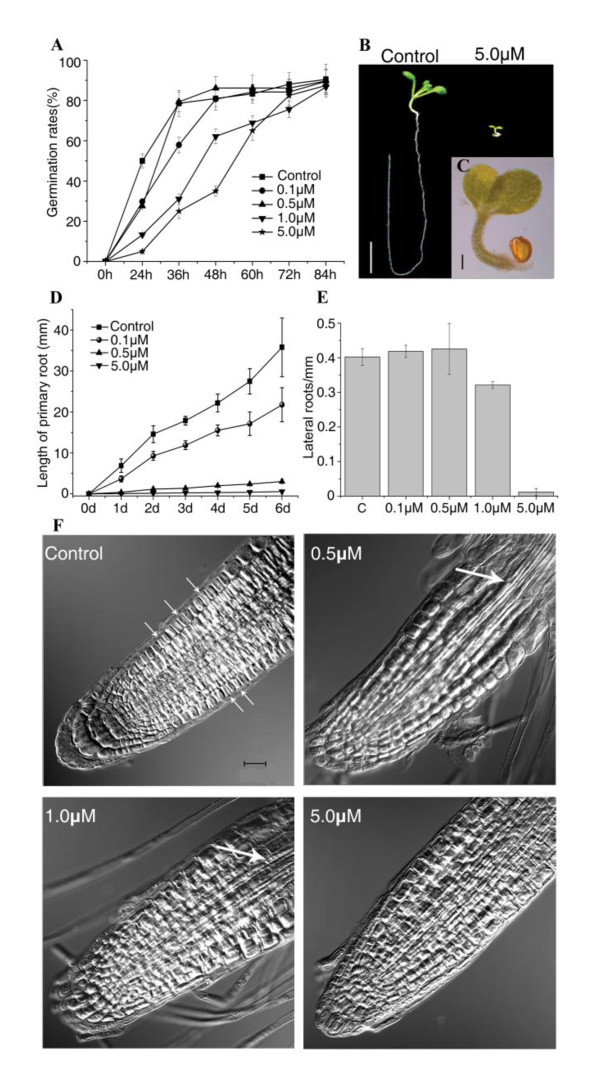
**Effects of NCS on the post-embryonic development of *Arabidopsis *roots**. (A) Inhibitory effect of NCS on radicle protrusion during seed germination. (B) Wild type *Arabidopsis *seedling at 10 days after stratification grown on medium containing 0 or 5.0 μM NCS. Bar = 5 mm. (C) A close-up view of the whole plant in (B) treated with 5.0 μM NCS. Bar = 1 mm. (D) NCS inhibits primary root elongation. Root growth time course was measured after transferring 3-day-old *Arabidopsis *seedlings to NCS-containing medium. (E) NCS affects the lateral root density after exposing to NCS for 7 days. Data shown in (A), (D), (E) are means ± SD obtained from three independent experiments (n ≧ 20, P < 0.01). (F) Effects of NCS on cell expansion and cell division of meristematic cells. Arrows in the control panel showed the dividing cells. Bars = 50 μm in all panels. Differential interference contrast (DIC) images were captured using the transmission light detector of the confocal microscope.

To study the effects of NCS on the development of *Arabidopsis*, we first tested the effects of various concentrations of NCS on the germination rate of wild type (WT) *Arabidopsis *seeds. After 84 h continuous monitoring, we found that NCS significantly delayed the radicle emergence and seed germination. In the control medium, more than 80% of the *Arabidopsis *seeds germinated within 36 h, but in the medium containing 1.0 or 5.0 μM NCS only about 20% of the seeds germinated in 36 h (Figure 1A). In addition, the seedling growth was significantly inhibited by 5.0 μM NCS (Figure 1B and 1C). The root growth ceased after germination and primary roots only grew to about 1.0 to 2.0 mm in the presence of 5.0 μM NCS (Figure 1C). These results indicate that NCS inhibits radicle elongation.

To further dissect the role of NCS in postembryonic development, we examined the effects of NCS on the primary root elongation and lateral root density. As shown in Figure 1D, the primary root growth was inhibited by NCS at as low as 0.1 μM, and 5.0 μM NCS almost completely inhibited the primary root growth. The relative growth rate of primary roots was also greatly inhibited after transfer to NCS (Additional file 1: Figure S1A). The lateral root density was not affected by 0.1 or 0.5 μM NCS, but was inhibited about 21.2% by 1.0 μM NCS, and was nearly completely inhibited by 5.0 μM NCS (Figure 1E).

Plant morphogenesis relies on regulated cell division and directed cell expansion [29]. To examine whether NCS affects cell division or cell expansion, we examined cells in primary roots using confocal microscopy. As shown in Figure 1F and ^Q2^Additional file 1: Table S1, cells in the meristem of NSC-treated roots were larger than those in control roots, indicating NCS stimulates cell expansion in the meristem zone. The number of dividing cells in root tips was greatly decreased compared to the control (Figure 1F) and the mitotic index in the meristem zone of *Arabidopsis *roots was also reduced (Additional file 1: Figure S1B) after NCS treatment, suggesting that NCS inhibits cell proliferation in *Arabidopsis *roots. The results also showed that the vascular bundle gradually extended from the upper zone to the bottom part of roots after 0.5 and 1.0 μM NCS treatment (Figure 1F and Additional file 1: Figure S2). However, 5.0 μM NCS treatment did not show this phenotype. It means that low concentration of NCS may stimulate cell differentiation in root tips.

**Figure 2:**
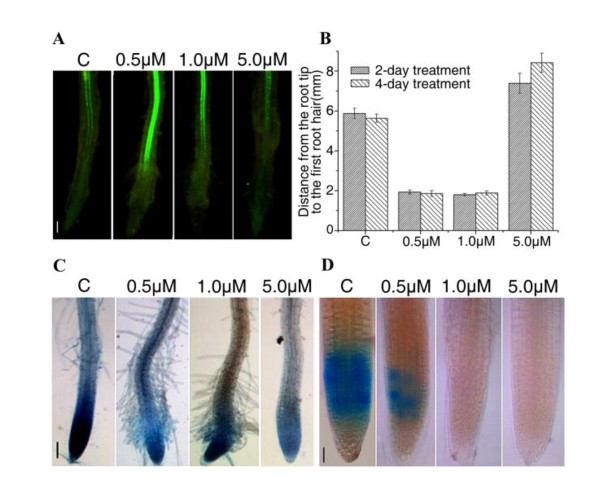
**Effects of NCS on cell division and cell differentiation in *Arabidopsis *root tips**. (A) NCS affects the differentiation of xylem pole pericycle cells in the root tip. Bar = 200 μm. (B) The development of root hairs in *Arabidopsis *primary roots was affected by NCS. Data shown are means ± SD obtained from three independent experiments (n ≧ 20). (C) *CDKA;1::uidA *promoter activity after NCS treatment for 4 days. Bar = 200 μm. (D) Expression of the mitotic marker *CYCB1;1::uidA *after 2 days NCS treatment. Bar = 50 μm.

To further confirm the effects of NCS on cell differentiation, the expression of selected *Arabidopsis *GAL4 enhancer trap transactivation line J0121 in the meristem zone was investigated. After 0.5 or 1.0 μM NCS treatment for 2 days, the expression of GFP increased in root tips, suggesting that the differentiation of pericycle cells was stimulated (Figure 2A). During the differentiation of vascular cells (Figure 1F and Additional file 1: Figure S2), root hairs emerged from epidermal cells in the apical region of NCS-treated roots but not control roots (Figure 2B and 2C), suggesting that NCS can stimulate the development of root hairs in *Arabidopsis *root tips. These observations suggest that 0.5 or 1.0 μM NCS may directly or indirectly stimulate cell differentiation in proliferating tissues. With 5.0 μM NCS treatment, the expression of GFP decreased in the apical region compared to the control (Figure 2A). In addition, the first root hair in 5.0 μM NCS treated roots was farther from the root tip than that in the control (Figure 2B). This suggests that the effects of NCS on cell differentiation in *Arabidopsis *roots are concentration-dependent.

When cells reach a predetermined size, cell fate (division, differentiation or other processes) is governed by various cell-cycle control proteins in concert with diverse signals [30]. To examine how NCS affects the process of cell division, two GUS fusion lines, *CDKA;1::uidA *and *CYCB1:1::uidA*, were used. The *CDKA:1 *promoter activity reflects the state of competence for cell division [31,32] and is constitutively expressed throughout the cell cycle [11]. In contrast, the *CYCB1:1 *promoter activity marks the progression from late G2 to M phase [33]. Whole-mount GUS assay samples were prepared from each line at day 2 or day 4 after transferring to the NCS-containing medium. The expression of *CDKA;1 *was inhibited after 4 days of 1.0 and 5.0 μM NCS treatment (Figure 2C). The *CYCB1:1 *promoter activity was also inhibited (Figure 2D) after 2 days of NCS treatment. These findings indicate that NCS can affect the expression of cell cycle genes in *Arabidopsis *roots.

We further investigated the expression of a quiescent center (QC) marker line, *QC25*. As shown in Additional file 1: Figure S3A, the expression of *QC25 *was not affected by NCS. When NCS-treated *Arabidopsis *seedlings were transferred to the NCS-free medium, the primary roots grew normally (Additional file 1: Figure S3B, C and D). Furthermore, NCS neither induced cell death (Additional file 1: Figure S4A) nor oxidative stress (Additional file 1: Figure S4B and C) in *Arabidopsis *roots, indicating that this substance did not exert toxic effects on *Arabidopsis *roots at concentrations used in this study.

### Effects of NCS cannot be rescued by hormone treatment

Phytohormones exert profound effects on plant growth and development. To investigate whether the inhibited *Arabidopsis *root growth is caused by lack of a hormone in the medium containing NCS, we supplemented the medium with a variety of phytohormones: NAA, IAA, 2,4-D, 6-BA, KIN and GA (each was applied at concentrations of 10^-9^, 10^-8^, 10^-7^, 10^-6 ^and 10^-5 ^M). Results showed that none of these phytohormones were able to restore the root growth in either 0.5 or 5.0 μM NCS treatments (Additional file 1: Table S2). Thus, we concluded that the inhibitory effects of NCS on *Arabidopsis *root growth were not caused by deficiency of any of these growth regulators.

### Effects of NCS on cell cycle progression

To specifically study the effects of NCS on cell cycle progression, a suspension cell system that allows cell synchronization with inhibitors of cell cycle progression should be used. For this reason, we chose the highly specialized tobacco (*Nicotiana tabacum*) cell line (BY-2) that has been extensively used for cell cycle studies in plants [34].

**Figure 3:**
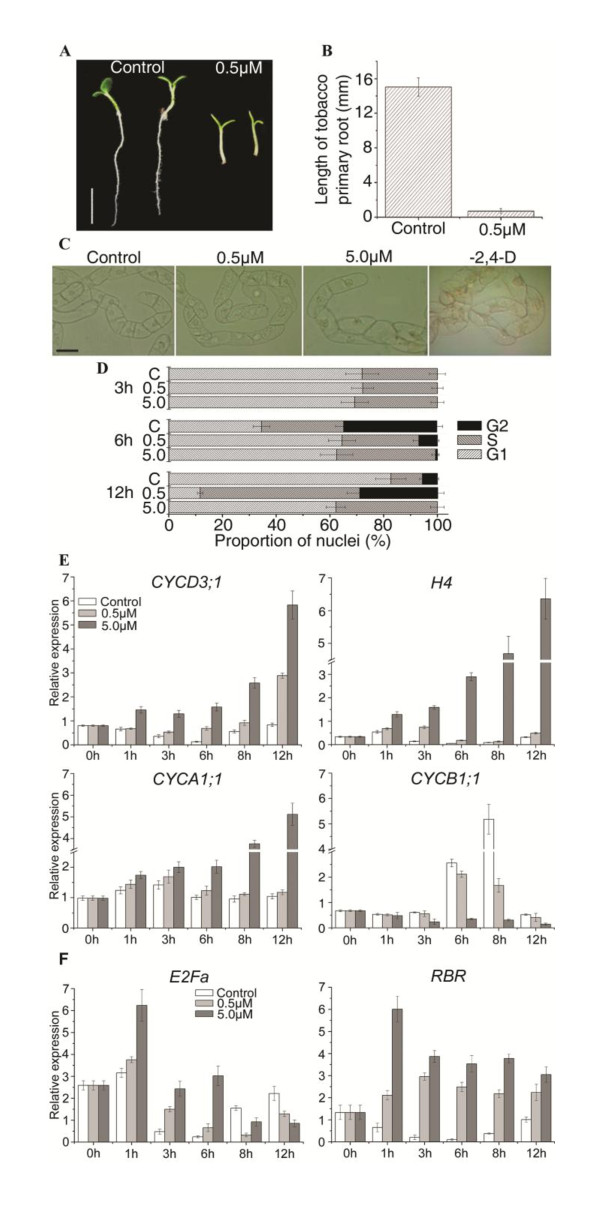
**Using a tobacco cell suspension to determine the effects of NCS on cell cycle**. (A) The phenotype of 7 days after germination (DAG) tobacco seedlings grown on 0.5 μM NCS. Bar = 5 mm. (B) Primary root length of 7 DAG tobacco seedlings was inhibited by NCS. Values are means ± SD of three independent experiments (n ≧ 20). (C) Images of BY-2 cells treated with NCS and depletion of 2,4-D for 2 days. Bar = 50 μm. (D) Flow cytometric analysis of the cell cycle. (E) qRT-PCR detection of *CYCD3;1, Histone H4*, *CYCA1;1 *and *CYCB1;1 *mRNA after NCS-treatment in synchronized tobacco BY-2 cells. (F) *E2Fa *and *RBR *transcription level in BY-2 cells after NCS-treatment. The relative expression is normalized to *ACTIN2*. Data shown were the means ± S.D of three independent experiments.

First, whether NCS had similar inhibitory effects on tobacco root development as observed in *Arabidopsis *roots was determined by germinating tobacco seeds on a medium containing 0.5 μM NCS. Similar to the observation in *Arabidopsis *plants, NCS significantly inhibited the primary root growth of tobacco seedlings (Figure 3A and 3B). Then, we examined whether NCS treatment affects cell division in BY-2 suspension cultured cells. Exponentially growing BY-2 cells were washed with medium lacking 2,4-D for three to five times, then equal amount of cells were diluted in different media. After two-day treatment, cells were photographed. As shown in Figure 3C, chains of small BY-2 cells were observed in the control medium. Although cell division was not completely inhibited, 0.5 μM NCS-treated cells were bigger than that of the control (Figure 3C). When treated with 5.0 μM NCS, most BY-2 cells greatly expanded similar to auxin-starved cells (Figure 3C). The effect of NCS treatment on cell cycle progression in highly synchronized BY-2 cells was further validated using flow cytometric analysis. BY-2 cells were treated with different concentrations of NCS after depletion of **a**phidicolin (see methods below). There was no difference between control and NCS-treated cells in 3 h, suggesting that NCS did not affect cells entry into S phase after depletion of aphidicolin (Figure 3D). Six hours after depletion of aphidicolin, 35.6% of control cells were at G2 phase, whereas only 7.3% of 0.5 μM NCS-treated cells and 0.7% of 5.0 μM NCS-treated cells were at G2 phase. After 0.5 μM NCS treatment for 12 h, a pronounced increase of the nuclear population at S phase was observed (Figure 3D). The population of S phase nuclei remained at about 35.2% after 5.0 μM NCS treatment for 3 h, and did not change up to 12 h (Figure 3D). The almost complete depletion of the G2 population after 5.0 μM NCS treatment indicated that few cells could pass S phase to G2 phase under this condition. To study the underlying mechanism of cell cycle blocking induced by NCS treatment, the expression of four tobacco cell cycle genes, *CYCD3:1*, *Histone H4*, *CYCA1:1 *and *CYCB1:1 *was investigated by quantitative reverse transcription PCR (qRT-PCR). Results in Figure 3Eshowed that in untreated synchronized cells, *CYCD3:1 *mRNA accumulated at the early G1 phase at 0 h and down-regulated during progression through the S phase; *Histone H4 *mRNA accumulated at the G1-to-S phase transition from 0 to 1 h, whereas *CYCA1:1 *mRNA started to accumulate at mid-S phase from 3 to 6 h. The expression of *CYCB1:1 *increased at G2-to-M phase from 6 to 8 h. The expression pattern of these four cyclin genes was similar to that previously observed [35], confirming the marker role of each gene in the cell cycle transition. Interestingly, the temporal expression pattern of cell cycle genes described above was altered by NCS treatment. 0.5 μM NCS increased the expression levels of *CYCD3:1*, *Histone H4*, and *CYCA1:1 *at specific phases but delayed the timing of expression (Figure 3E). For *CYCB1:1*, 0.5 μM NCS markedly modified the amplitude at 8 h. However, 5.0 μM NCS greatly stimulated the expression of *CYCD3:1*, *Histone H4 *and *CYCA1:1*, and disturbed the pattern of expression compared to the control. The expression of *CYCB1:1 *was greatly inhibited by 5.0 μM NCS. These results showed that the expression of cell cycle genes in BY-2 cells was disturbed by NCS. To further investigate the effects of NCS on the cell cycle gene expression and DNA replication, we examined the mRNA levels of *E2Fa *and *RBR *in synchronized BY-2 cells. The *E2Fa *transcripts were up-regulated from 1 to 6 h, and down-regulated from 8 to 12 h of NCS treatment (Figure 3F). With the NCS treatment for 1-12 h, *RBR *transcripts were significantly stimulated (Figure 3F), confirming that the transcription activity of E2Fa/DPa on the gene transcription and entry into S phase may be repressed by NCS through up-regulation of *RBR *expression.

### NCS affects the expression of core cell cycle genes in *Arabidopsis *root tips

**Figure 4:**
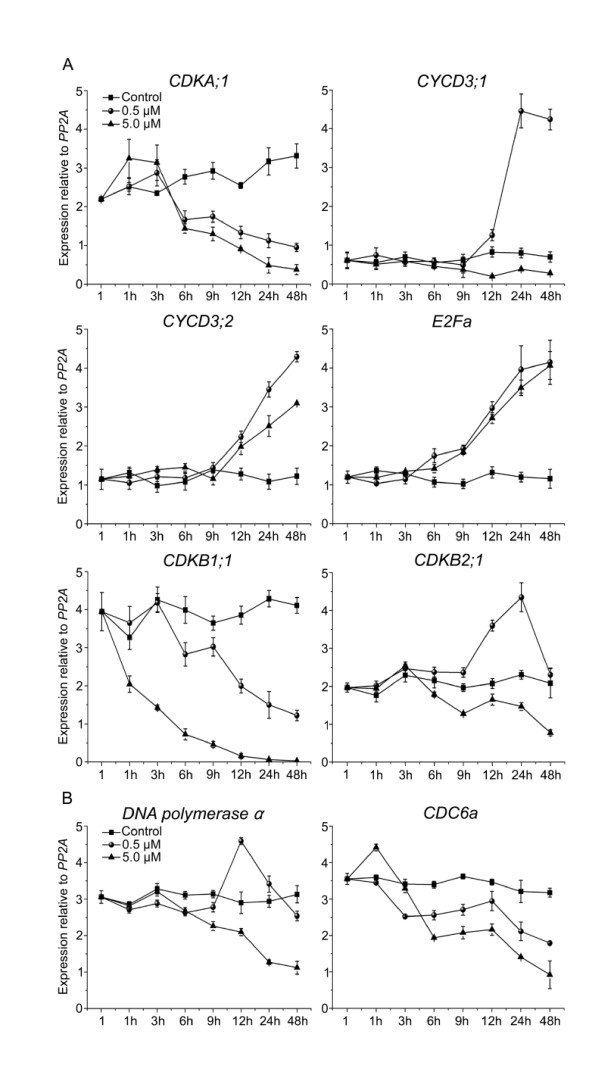
**qRT-PCR analysis of the expression of core cell cycle genes in *Arabidopsis *root tips**. (A) qRT-PCR results showing the effects of NCS on expression of *CDKA;1*, *CYCD3;1*, *CYCD3;2*, *E2Fa*, *CDKB1;1 *and *CDKB2;1 *in *Arabidopsis *root tips. (B) The relative expression (normalized to *PP2A*) of *DNA polymerase α *and *CDC6a *after NCS treatment for indicated times. Data shown in (A) and (B) are means ± SD of three independent experiments.

To further validate our studies on the expression of cell cycle genes in BY-2 cells, we analyzed the effects of NCS on mRNA levels of key cell cycle genes in root tips of *Arabidopsis *seedlings by qRT-PCR. Plants were sampled at eight time points during treatment to check the expression of *CDKA;1*, *CYCD3:1*, *CYCD3:2*, *E2Fa*, *CDKB1:1 *and *CDKB2:1 *(Figure 4A). *CDKA;1 *transcript was inhibited by NCS after 6 h treatment. Expression of *CYCD3:1 *was increased sharply from 9 h of 0.5 μM NCS treatment. *CYCD3:2 *showed a very similar expression pattern as *CYCD3:1 *in 0.5 μM NSC treatment. However, unlike *CYCD3:1*, an increase in *CYCD3:2 *expression was also observed in 5.0 μM NCS treatment. As shown in Figure 4A, 0.5 and 5.0 μM NCS enhanced *E2Fa *mRNA accumulation. The expression of *CDKB2:1 *increased about two fold at 12 and 24 h, and then decreased to the control level at 48 h in 0.5 μM NCS treatment, whereas in 5.0 μM NCS treatment, the expression was inhibited after 6 h. *CDKB1:1 *transcript levels steadily decreased in plants treated with NCS (Figure 4A). These results indicated that the expression of G1/S and G2/M phase gene in *Arabidopsis *root tips was disturbed by NCS. This perturbation may affect the cell differentiation in *Arabidopsis *root tips.

Previous studies suggest that cell division and differentiation are accompanied by full genome duplication [36]. In this study, we showed that 0.5 and 1.0 μM NCS treatment stimulated cell differentiation in *Arabidopsis *roots (Figure 1F, 2B and 2C). The differentiation of pericycle cells and root hairs was inhibited by 5.0 μM NCS, although the expression of *CYCD3:2 *was increased. In order to test whether the effects of 5.0 μM NCS on cell differentiation were caused by inhibition of DNA replication, we further analyzed two putative E2F targets, *CDC6a *and *DNA polymerase α*, which contain E2F sites in their promoters and participate in the initiation of DNA replication [37]. The result showed that *DNA polymerase α *exhibited a similar changing pattern as *CDKB2:1*, i.e. the *DNA polymerase α *transcription was stimulated by 0.5 μM NCS at 12 h, but inhibited at 48 h, and was dramatically decreased by 5.0 μM NCS treatment (Figure 4B). *CDC6a *mRNA was greatly decreased by 5.0 μM NCS, but was only slightly inhibited by 0.5 μM NCS treatment before 12 h (Figure 4B). This means that the E2Fa/DPa activity was not affected by 0.5 μM NCS before 12 h. Down-regulation of these two genes by 5.0 μM NCS affects the formation of origin recognition complex (ORC), and then disturbs the activation of DNA replication origins. This finding indicates that NCS may affect the transcription activity of E2Fa/DPa in a concentration-dependent manner.

### Reduction of CDK activity in NCS-treated *Arabidopsis *roots

**Figure 5:**
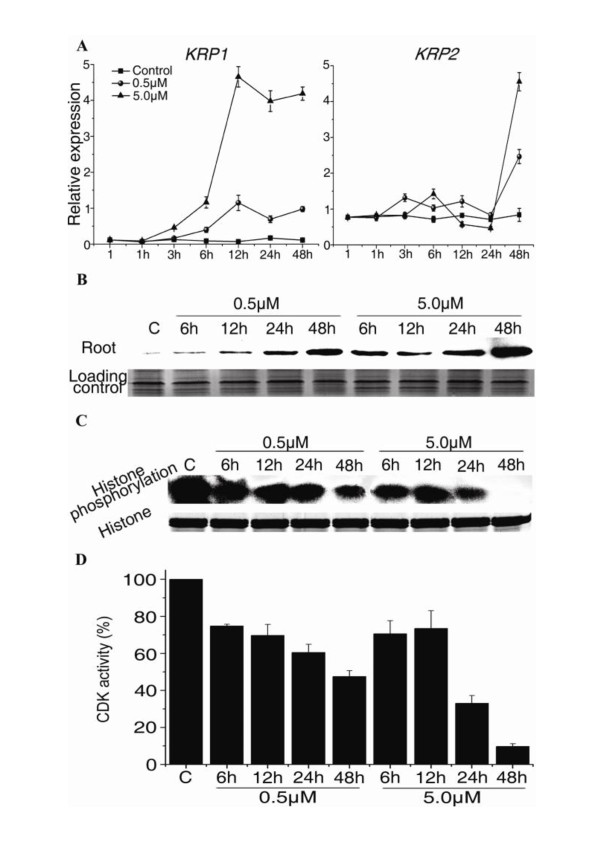
**Analysis of *KRP1 *and *2 *expression and CDK activity after NCS treatment**. (A) qRT-PCR analysis of *KRP1 *and *KRP2 *expression relative to *PP2A*. (B) Immunoblot analysis of NCS-treated *Arabidopsis *roots using an anti-KRP2 antibody. Coomassie Brilliant Blue staining of the electrophoresis gel was used as a loading control. (C) P10^CKS1At^-associated kinase activity in NCS-treated roots. Autoradiogram showing typical results of CDK activity assays with histone as the substrate. Coomassie Brilliant Blue staining of the electrophoresis gel with histone was used as a control for equal substrate quantity per phosphorylation reaction. (D) Relative quantification of three independent kinase activity measurements as depicted in (C). The control was arbitrarily set at 100%. Data shown in (A) and (D) are means ± SD of three independent experiments.

As previously reported, the CDKA activity determines whether a cell undergoes division or differentiation [13,38]. To analyze the effects of NCS on CDKs, we examined the expression of two CDK inhibitors, *KRP1 *and *KRP2*, by qRT-PCR. *KRP1 *mRNA was mildly upregulated by 0.5 μM NCS. In contrast, it was strongly stimulated in 5.0 μM NCS-treated roots (Figure 5A). The *KRP2 *expression pattern in NCS-treated roots was different from that of *KRP1*. The expression of *KRP2 *was not affected by NCS before 24 h, but was stimulated at 48 h of NCS treatment (Figure 5A).

KRP1 and KRP2 protein abundance is regulated post-transcriptionally through CDK phosphorylation and proteasomal degradation [39]. Therefore, we checked the KRP2 protein abundance by immunoblot analysis. Differences of KRP2 protein abundance between roots treated with 0.5 and 5.0 μM NCS were observed (Figure 5B). The level of KRP2 in 0.5 μM NCS-treated roots was elevated at 48 h. In 5.0 μM NCS-treated roots, KRP2 accumulated after 6 h of treatment and was higher at 6, 12, 24 and 48 h than that in 0.5 μM NCS-treated roots (Figure 5B).

To further determine whether upregulation of these two inhibitors affects the CDK activity, we measured the p10CKS1At-bound CDK activity in *Arabidopsis *roots. The CDK activity was slightly inhibited by NCS before 12 h, but it decreased to 45.5% of the control in 0.5 μM NCS treatment for 48 h and to 33.2% and 10.1% in 5.0 μM NCS treatment for 24 and 48 h, respectively (Figure 5C and 5D). The CDK activity was more severely inhibited at 24 and 48 h in 5.0 μM NCS-treated roots than in roots treated with 0.5 μM NCS (Figure 5C and 5D). It has been postulated that CDKB1;1 regulates the CDKA;1 activity in dividing cells through controlling the KRP2 protein abundance [39]. If so, the accumulation of KRP2 protein and the inhibition of the CDK activity observed following NCS treatment may be caused by the inhibition of *CDKB1;1 *expression. Together with the data on cell differentiation and expression of *E2Fa *downstream genes in *Arabidopsis *root tips, these results indicate that CDK/cyclin complexes with roles in both cell division and differentiation are impaired by 5.0 μM NCS treatment, whereas the mitotic CDK complexes are mainly targeted by NCS at 0.5 μM.

## Discussion

### NCS inhibits cell proliferation in the root apical meristem

Germination entails the resumption of growth and development by a complex series of processes and is generally considered to be complete when the radicle penetrates the seed coat [40]. In the germination process, exit from G1 phase, entry into S phase, and cell division in a subset of cells in the root meristem precedes radicle protrusion in *Arabidopsis *[41]. In the present study, we found that NCS significantly delayed radicle emergence (Figure 1A). Similar effects of aphidicolin and roscovitine were observed in previous research. As a DNA polymerase and CDK inhibitor, respectively, aphidicolin and roscovitine significantly delay radicle protrusion, indicating that both DNA synthesis and CDK activity contribute to the elongation of the radicle during its emergence [41]. These findings suggest that cell division is important for radical emergence. Our data also showed that cell division (Figure 1F), mitotic index (Additional file 1: Figure S1B) and the expression of G2/M phase cell cycle genes in *Arabidopsis *roots were inhibited by NCS (Figure 2D and 4A). The p10^CKS1At^-bound CDK activity in *Arabidopsis *roots was reduced by NCS (Figure 5C and 5D). All these results strongly suggest that the effects of NCS on the post-embryonic development of *Arabidopsis *roots are correlated with reduced numbers of dividing cells in the radicle meristem zone.

### NCS modulates the expression of cell cycle genes

Cell cycle regulation is of pivotal importance for plant growth and development. Studies have shown that a key element of the cell cycle progression control in plants is the regulation of cell cycle genes [5-7,42]. For example, Dewitte et al. found that constitutive over-expression of *CYCD3:1 *increased the CYCD3:1-associated kinase activity, reduced the proportion of cells in the G1 phase of the cell cycle, and caused marked developmental changes [7]. In addition, ectopic expression of *E2Fa *induced sustained cell proliferation in normally-differentiated cotyledons and hypocotyls [43]. Boudolf et al. found that the expression of a dominant-negative allele of the *Arabidopsis CDKB1:1 *gene inhibited cell division and enhanced endo-reduplication [44]. In the present study, the expression of some cell cycle genes in both BY-2 cells (Figure 3E and 3F) and *Arabidopsis *root tips (Figure 4A) was affected by NCS. In general, the expression of G1/S phase genes was stimulated but the expression of G2/M phase genes was reduced by NCS treatment. For some cell cycle genes, such as *CYCD3;1*, the effect of NCS on its expression is concentration-dependent in *Arabidopsis *root tips (Figure 4A). There are also some discrepancies in the effects of NCS on cell cycle genes in synchronized BY-2 cells and *Arabidopsis *root tips. For example, the mRNA level of *CYCD3;1 *was differently affected by 5 μM NCS (Figure 3E and 4A). This may be caused by the differences between the two plant cell systems. Previous results also showed that the expression of D-type cyclins in BY-2 cell cultures is different from that in *Arabidopsis *cell cultures (6).

We further found that NCS stimulated the transcript accumulation of *KRP1*, and the KRP2 protein abundance in *Arabidopsis *roots (Figure 5A and 5B). With the function of these two inhibitors, the CDK activity was affected differently by NCS depending on concentrations. The decreased CDK activity affected the transcriptional activity of E2Fa/DPa (Figure 4B). This also explains why the expression of DNA polymerase α was up-regulated by 0.5 μM NCS at 12 h. The transcriptional activity of E2Fa/DPa may not be inhibited by 0.5 μM NCS at 12 h because the CDK activity was not low enough (Figure 5C and 5D). The disturbance of expression of G1/S and G2/M phase cell cycle genes by NCS significantly affected the post-embryonic development of the *Arabidopsis *roots.

### NCS shows different roles in cell differentiation through KRPs in a concentration-dependent manner

As proven in other eukaryotic organisms, cyclin-dependent kinases (CDKs) govern the cell cycle in plants. Different CDK-cyclin complexes phosphorylate a plethora of substrates at the key G1-to-S and G2-to-M transition points, triggering the onset of DNA replication and mitosis, respectively. In plants, a bona fide PSTAIRE CDK, designated CDKA, plays a pivotal role at both the G1-to-S and G2-to-M transition points. Overproduction of a dominant negative *CDKA *of *Arabidopsis thaliana *in tobacco plants yields smaller plants. However, the G1/G2 ratio remains unaltered, corresponding with the observation that the CDKA activity can be detected at both checkpoints [45-47]. Thus, CDKA is essential at both G1-to-S and G2-to-M transitions of the cell cycle. Plants also possess a unique class of CDKs, the so called B-type CDKs that have not been described in any other organisms [48-50]. In B-type CDKs the PSTAIRE hallmark presented in CDKAs is replaced by either PPTALRE or PPTTLRE, reflecting the existence of two subgroups, CDKB1 and CDKB2 [14]. The requirement of CDKB1 activity to progress through mitosis has been demonstrated with a dominant negative approach, illustrating that a reduction in the CDKB1 activity results in an increased 4C/2C ratio because of a block at the G2-to-M transition [47,51]. Our results showed that the CDK activity was differently affected by NCS in *Arabidopsis *roots (Figure 5C and 5D). Although the p10^CKS1At^-bound CDKs probably contain both A-type and B-type CDKs, the decreased CDK activity at 6 and 12 h inhibited cell division (Figure 1F and Additional file 1: Figure S1). It means that the G2/M transition needs a higher CDK activity than the G1/S transition. Previous study showed that in *Drosophila *a special CDK inhibitor, ROUGHEX (RUX), binds to and inactivates mitotic CDK complexes, helping to establish a G1-phase with low CDK activity [52,53]. In addition, onset of S phase requires a lower threshold of protein kinase activity than onset of mitosis, ensuring that a G1 cell would automatically initiate S phase before mitosis [54]. From this viewpoint, the role of 0.5 or 1.0 μM NCS on cell differentiation could be an indirect effect, whereby the inhibitory effects of low concentrations of NCS on cell cycle in mitotically dividing cells may trigger the initiation of cell differentiation in the *Arabidopsi*s root tips.

In several mis-expression studies, KRPs block endo-replication and reduce cell numbers, leading to dwarfed plants in extreme cases [13,17,18,39,55,56]. We observed similar phenomena in *Arabidopsis *roots, since the expression of *KRP1 *and the abundance of KRP2 protein were enhanced by NCS (Figure 5A and 5B). However, the *KRP2 *transcript was not stimulated by NCS before 24 h. It means that the *KRP1 *expression is more sensitive to NCS than that of *KRP2*, and that the inhibitory effects of NCS at the first few hours may be caused by the upregulation of *KRP1*. From this point of view, stimulation of *KRP1 *may be the reason why NCS inhibits cell division at the first few hours. To verify this, we checked the KRP2 protein abundance after NCS treatment (Figure 5B). The result indicated that upregulation of *KRP1 *caused the accumulation of KRP2 protein by its role on the CDK activity before 24 h. Previous results of mis-expression of *KRP1 *observed by Weinl et al. suggested that *KRP1 *acts in a concentration-dependent manner, blocking the G1/S transition at high concentrations, and the G2/M transition at low concentrations [56]. In addition, Verkest et al. reported that KRP2 gain-of-function has either a positive or negative effect on plants' DNA ploidy levels, depending on the level of *KRP2 *over-expression [39]. Our results showed that 0.5 μM NCS mildly upregulated *KRP1 *(Figure 5A) and KRP2 (Figure 5B), thus causing prematuration in the meristematic zone (Figure 1F, Additional file 1: Figure S2 and Figure 2A). In contrast, 5.0 μM NCS significantly stimulated the *KRP1 *expression (Figure 5A) and KRP2 protein abundance (Figure 5B), seriously inhibiting the CDK activity which is required for the progression of G1/S phase at 24 and 48 h (Figure 5C and 5D), and then inhibited cell differentiation in *Arabidopsis *root tips.

In higher plants, where organogenesis occurs continuously, most cells maintain their ability to re-enter and regulate the cell cycle in response to molecular signals, such as auxin. Previous report showed that the expression of *KRP1 *and *KRP2 *is high in the inactive pericycle cells of NPA-treated roots [32]. Our recent results showed that polar auxin transport in *Arabidopsis *roots was inhibited by NCS [57]. It means that the effects of NCS on cell cycle progression may be caused by its role on auxin transport. We also showed that exogenous plant hormones cannot restore the inhibitory effects of NCS on root development (Additional file 1: Table S2), which is different from that of NPA, a general auxin polar transport blocker [58]. The treatment with exogenous hormones may not rescue the state of hormone distribution and/or signaling in NCS treated roots. The effects of NCS on the development of root hairs may be also caused by its role on auxin signaling, because previous research confirmed that auxin participates in the differentiation of root hairs [59]. However, how NCS affects auxin signaling in *Arabidopsis *roots remains to be elucidated.

## Conclusions

Taken together with earlier findings, our results indicate that at low concentration NCS preferentially inhibits mitotic cell cycle specific/cyclin complexes, whereas at high concentration the strong accumulation of KRP1 and KRP2 protein affects the CDK/cyclin complexes with consequent effects at both G1/S and G2/M phases. Thus, the inhibition effects of low concentration of NCS on cell cycle in mitotically dividing cells may trigger the initiation of cell differentiation in *Arabidopsis *root tips. This disturbance of the balance of G1/S and G2/M phase cell cycle genes by NCS significantly affected the post-embryonic development of the *Arabidopsis *roots. Identifying the molecular targets in the plant cell of this natural compound may provide new insights about the regulatory pathways involved in cell cycle control and cell differentiation in the root meristem.

## Methods

### Purification of NCS

NCS was isolated and purified from *N. tazetta *bulbs according to Bi et al. [27].

### Plant materials and culture conditions

The *Arabidopsis *Columbia ecotype (Col-0), and the transgenic lines *CYCB1:1::uidA*, *CDKA;1::uidA *(C24), *QC25::uidA*, and J0121 were used in this study. Initially, the effects of NCS on the germination rate of WT *Arabidopsis *seeds and the root growth of resulting seedlings were examined by surface-sterilizing seeds and placing them on plates of 1/2 × MS medium (pH 5.7) containing 1.0% (w/v) sucrose, 1.0% (w/v) agar and NCS at concentrations of 0.1, 0.5, 1.0 or 5.0 μM. The plates were kept at 4°C for 4 days before transferring to a growth chamber, where they were placed in racks at an angle of ca. 85° relative to horizon. The plates were maintained at 21 ~ 23°C under a 16/8 h photoperiod for 84 h, during which they were continuously observed and their germination rates and timing of radical emergence were recorded. After transferring the three-day-old seedlings to NCS for 7 days, the seedlings were photographed and the length of their primary roots was measured from digital images of the plates using Image J software (NIH, version 1.62), while lateral roots and lateral root primordia were counted under a dissecting microscope.

### Tobacco BY-2 cell suspension and synchronization

Tobacco Bright Yellow 2 (BY-2) cell cultures were maintained as described [34]. For experiments with exponentially growing BY-2 cells, 3-day-old suspensions were used. For experiments with synchronized cells, synchronization was carried out as described by Reichheld. Briefly, sequential treatment with 3 μg ml^-1 ^aphidicolin (Sigma, USA) for 24 h was used to specifically study the G1-to-S phase transition [35]. For the treatment with NCS at the G1-to-S phase transition, NCS was added at the indicated concentrations 1 h after the removal of aphidicolin. The time point of adding NCS was designated time 0 of the experiment.

### Flow cytometry

For flow cytometric analysis, protoplasts of tobacco BY-2 cells were prepared by incubating cells for 1 h with 1.5% Cellulase R-10 (Yakult Company, Japan) and 0.1% Mcerozyme R-10 (Yakult Company, Japan). The cells were incubated at 27°C, washed, and lysed in Galbraith's buffer [60], filtered in 1% formaldehyde through 10 μm nylon mesh, treated with RNase A, and stained with propidium iodide (50 μg ml^-1^). Cytometric analysis was performed using at least 10^4 ^nuclei on an EPICS flow cytometer (Beckman Coulter, USA). For all results presented in the text, two populations were considered as significantly different if their deviation exceeded 5%.

### Confocal microscopy

Confocal images were captured with a LSM510 Laser Confocal Scanning Microscope (Zeiss, Jena, Germany) using argon laser excitation at 488 nm and a 505-550 nm emission filter set for GFP fluorescence observation.

### Histochemical analyses

Histochemical assays of the GUS activity were performed as described by Jefferson et al. with minor modifications [61]. Three-day-old *CYCB1:1::uidA*, *CDKA;1::uidA *or QC25 seedlings were treated with NCS (at concentrations and for times indicated in the text and the figures), then submerged in GUS staining buffer containing 1 mM X-Gluc, 100 mM sodium phosphate (pH7.5), 0.5 mM potassium ferricyanide, 0.5 mM potassium ferrocyanide, 10 mM EDTA and 0.1% Triton X-100. Tissues were incubated at 37°C for 12 h and then fixed in 70% (v/v) ethanol. Starch granules were stained as described by Willemsen et al. [62]. Samples were mounted in 30% glycerol and photographed under a dissecting microscope.

### Quantitative reverse transcription PCR analysis

Total RNA was extracted with Trizol (Invitrogen) from 100 mg samples of cells of the tobacco line BY-2, or from ca. 5 mm samples of excised root segments and root tips of 6-day-old WT *Arabidopsis *seedlings. Before PCR analysis, total RNA was pretreated with RNase-free DNase (Promega, USA) to eliminate any contaminating genomic DNA. First-strand cDNA was synthesized from 1 to 2 μg portions of total RNA using Superscript II reverse transcriptase (Invitrogen, USA). PCR reactions of 20 μl were prepared using the Takara SYBR Premix ExTaq, with 2 μl of template DNA. Real-time thermo cycling was performed using a ROTOR-GENE 3000 instrument (Corbett, Australia) with the following standard cycling conditions: 95°C for 10 s, followed by 40 cycles of 95°C for 5 s and 60°C for 30 s. The results were analyzed by Rotor-Gene Real-Time Analysis Software 6.1 (Build 81). The specific primers for each gene are shown in Additional file 1: Table S1.

### Protein extraction and immunoblotting

Roots of 6-day-old *Arabidopsis *plants were harvested and used immediately, or frozen in liquid nitrogen and stored at -70°C. Proteins were extracted by grinding tissues with quartz sand in homogenization buffer (50 mM Tris, pH 7.2, 60 mM β-glycerophosphate, 15 mM nitrophenyl phosphate, 15 mM EGTA, 15 mM MgCl_2_, 2 mM dithithreitol, 0.1 mM vanadate, 50 mM NaF, 20 μg ml^-1 ^leupetin, 20 μg ml^-1 ^aprotein, 100 μM benzamidine, 1 mM phenylmethylsulfonyl fluoride, and 0.1% Triton X-100). Following centrifugation at 10,000 g for 30 min, proteins in the supernatant were separated by 11.5% SDS-PAGE and blotted onto Immobilon-P membranes (Millipore, USA). Filters were blocked by immersing in 50 mM Tris, pH 7.4, 150 mM NaCl, and 0.05% Tween 20 (blocking buffer) containing 5% (w/v) powdered milk for 3 h at room temperature, and then incubated overnight at 4°C with KRP2 (1/1000) antibody in blocking buffer containing 1% (w/v) powdered milk. Antigen-antibody complexes were detected with horseradish peroxidase-conjugated IgG diluted 1/5000 with an ECL chemiluminescence system.

### In vitro kinase assay

P10^CKS1At^-bound CDK activity was measured as described by De Veylder et al. [63] with modifications. CKS1At was purified from an overproducing strain of *E. coli *and linked to CNBr-Sepharose 4B at a concentration of 11 mg protein per 1 g beads according to suppliers' instructions. Portions (50 μl) of the resulting suspension of p10^CKS1At^-Sepharose beads were washed with bead buffer (50 mM Tris, pH 7.4, 5 mM NaF, 250 mM NaCl, 5 mM EDTA, 5 mM EGTA, 0.1% Nonidet P40, 20 μg ml^-1 ^leupetin, 20 μg ml^-1 ^aprotein, 100 μM benzamidine and 1 mM phenylmethylsulfonyl fluoride) and mixed with portions of the extracts, prepared as described above, containing 150 μg protein in tubes that were then rotated constantly at 4°C for 3 h. After a brief centrifugation at 5000 rpm and removal of the supernatant, the beads were carefully washed three times with bead buffer, once with kinase buffer (50 mM HEPES, pH 7.5, 10 mM MgCl_2_, 1 mM dithiothreitol), and then used for kinase assays, as described by Hemerly et al. [45], with histone as CDK substrate.

### Accession number

*Nicotiana tabacum*: [*Actin2*:U60495, *CYCD3;1*:AB015222, *E2Fa*:AB025347, *H4*:AB280787, *CYCA1;1*:D50735, *CYCB1;1*:Z37978, *RBR*:AB015221]

*Arabidopsis thaliana*: [*PP2A*:AY099760, *CDKA;1*:AB009399, *CYCD3;1*:NM_119579, *CYCD3;2*:NM_126126, *CDKB1;1*:D10851, *CDKB2;1*:NM_106304, *E2Fa*:AJ294534, *DNA polymerase a*:NM_126110, *CDC6a*:NM_128522, *KRP1*:NM_127907, *KRP2*:NM_114923]

## Abbreviations

CDK: Cyclin-dependent kinases; KRP1: Kip-related protein1; KRP2: Kip-related protein2; MS: Murashige-Skoog; NCS: Narciclasine; ORC: Origin recognition complex; RBR: Retinoblastoma-related; RB: Retinoblastoma; qRT-PCR: Quantitative reverse transcription PCR; QC: Quite center; WT: Wild type.

## Competing interests

The authors declare that they have no competing interests.

## Authors' contributions

XN designed the studies, participated in the experiments and drafted the manuscript. YH carried out the immunoassays and histochemical analyses. KY participated in the q-RT-PCR. HL participated in the flow cytometry analysis. PJ helped to use the confocal microscope. HW and XW gave great suggestions for experiments and manuscript. YB conceived of the study, and participated in its design and coordination and helped to draft the manuscript. All authors read and approved the final manuscript.

## Supplementary Material

Additional file 1 **Table S1**. Effects of NCS on epidermal cell size in *Arabidopsis *root. **Table S2**. Effects of phytohormones and NCS on primary root growth of *Arabidopsis*. **Table S3**. List of PCR primers used in the present study. **Figure S1**. Effects of NCS on relative growth rate and mitotic index of *Arabidopsis *root. **Figure S2**. Effects of NCS on cell differentiation of vascular cells. **Figure S3**. Recovery of the inhibition effects of NCS on root development. **Figure S4**. Physiological effects of NCS in *Arabidopsis *root. Click here for file
