# Supplementary material for: Concentration-dependent effects of narciclasine on cell cycle progression in Arabidopsis root tips
Source: BMC Plant Biol. 2011 Dec 28;11:184. doi: 10.1186/1471-2229-11-184 (PMC3282671; doi:10.1186/1471-2229-11-184)
Supplement: Additional file 1 — Table S1. Effects of NCS on epidermal cell size in Arabidopsis root. Table S2. Effects of phytohormones and NCS on primary root growth of Arabidopsis. Table S3. List of PCR primers used in the present study. Figure S1. Effects of NCS on relative growth rate and mitotic index of Arabidopsis root. Figure S2. Effects of NCS on cell differentiation of vascular cells. Figure S3. Recovery of the inhibition effects of NCS on root development. Figure S4. Physiological effects of NCS in Arabidopsis root. [file 1471-2229-11-184-S1.DOC]

**Additional Material files**

**Concentration-dependent effects of narciclasine on cell cycle progression in the *Arabidopsis* root tip**

**Authors:** Xiaofan Na*, Yanfeng Hu*, Kun yue, Pengfei Jia, Hongxia Lu, Huahua Wang, Xiaoming Wang, Yurong Bi

School of Life Science, Lanzhou University, Lanzhou, 730000, People’s Republic of China

**Footnotes:**

* These authors contributed equally to this work.

| **Table S1.** Effects of NCS on epidermal cell size in *Arabidopsis* roots. The length and width of epidermal cells in *Arabidopsis* root meristem were measured after 7 days of NCS treatment. Results shown are the averages of 20 seedlings ± SD. P=0.01. | | |
| --- | --- | --- |
| NCS (μM) | cell length (μm) | cell width (μm) |
| 0 | 5.86 ± 0.99 | 9.86 ± 0.80 |
| 0.5 | 12.40 ± 1.56 | 11.14 ± 1.58 |
| 1.0 | 13.28 ± 3.01 | 10.28 ± 0.90 |
| 5.0 | 11.70 ± 2.50 | 10.64 ± 1.76 |
|  | | |
|
|

**Table S2 Effects of phytohormones and NCS on primary root growth of *Arabidopsis*. Absolute root growth was measured after 7 days of NCS treatment. Results shown are the averages of 20 seedlings ± SD. P=0.01.**

| NAA(M) | Concentration of NCS (μM) | | | | | | | |
| --- | --- | --- | --- | --- | --- | --- | --- | --- |
|  | 0 | | 0.5 | | 1.0 | | 5.0 | |
|  | root length(mm) | SD | root length(mm) | SD | root length(mm) | SD | root length(mm) | SD |
| 0 | 52.6 | 8.5 | 9.0 | 2.1 | 9.0 | 2.5 | 6.7 | 1.7 |
| 10^-9^ | 45.9 | 7.9 | 10.1 | 2.6 | 5.9 | 1.4 | 6.4 | 1.0 |
| 10^-8^ | 44.0 | 8.1 | 11.2 | 1.8 | 5.5 | 1.0 | 7.5 | 1.7 |
| 10^-7^ | 27.0 | 4.8 | 9.3 | 1.3 | 6.5 | 1.3 | 6.4 | 1.6 |
| 10^-6^ | 9.4 | 2.4 | 7.7 | 2.1 | 6.2 | 1.7 | 6.5 | 1.4 |
| 10^-5^ | 9.6 | 2.4 | 6.0 | 1.0 | 4.8 | 1.2 | 5.8 | 1.7 |
| IAA(M) |  | | | | | | | |
| 0 | 60.2 | 9.2 | 9.3 | 1.1 | 6.3 | 1.8 | 4.6 | 1.0 |
| 10^-9^ | 46.7 | 13.6 | 7.6 | 1.0 | 4.8 | 1.5 | 4.6 | 1.0 |
| 10^-8^ | 41.8 | 7.8 | 8.0 | 1.6 | 4.6 | 0.8 | 4.7 | 1.3 |
| 10^-7^ | 33.9 | 5.5 | 7.7 | 1.8 | 4.5 | 1.2 | 4.0 | 0.9 |
| 10^-6^ | 12.5 | 2.8 | 5.6 | 1.4 | 4.2 | 0.8 | 4.7 | 1.0 |
| 10^-5^ | 8.5 | 2.3 | 4.9 | 0.9 | 4.6 | 0.9 | 5.7 | 1.0 |
| 2,4-D(M) |  | | | | | | | |
| 0 | 44.7 | 10.6 | 6.5 | 1.2 | 4.2 | 0.6 | 2.3 | 0.6 |
| 10^-9^ | 40.8 | 7.6 | 6.9 | 1.5 | 3.7 | 0.8 | 3.0 | 0.6 |
| 10^-8^ | 30.8 | 9.7 | 5.7 | 1.4 | 3.6 | 1.0 | 2.3 | 0.4 |
| 10^-7^ | 5.9 | 1.7 | 4.8 | 1.2 | 3.7 | 0.9 | 3.1 | 0.7 |
| 10^-6^ | 4.5 | 1.0 | 3.7 | 0.9 | 2.9 | 0.5 | 3.1 | 0.7 |
| 10^-5^ | 4.0 | 0.6 | 3.8 | 0.7 | 2.8 | 0.5 | 2.8 | 0.5 |
| 6-BA(M) |  | | | | | | | |
| 0 | 46.1 | 8.4 | 6.6 | 1.5 | 5.0 | 1.3 | 4.7 | 1.2 |
| 10^-9^ | 45.2 | 12.0 | 8.3 | 1.9 | 5.2 | 1.3 | 4.6 | 1.3 |
| 10^-8^ | 44.2 | 5.9 | 6.4 | 2.2 | 5.6 | 1.4 | 4.8 | 1.3 |
| 10^-7^ | 30.0 | 6.4 | 9.4 | 3.2 | 6.4 | 2.1 | 4.7 | 1.4 |
| 10^-6^  10^-5^ | 19.0 | 3.4 | 7.4 | 2.2 | 7.1 | 2.0 | 5.0 | 1.3 |
|  | 14.2 | 2.3 | 7.1 | 2.0 | 6.2 | 1.5 | 4.4 | 1.1 |
| KIN | | | | | | | | |
| 0 | 41.6 | 7.8 | 6.7 | 1.9 | 5.1 | 1.1 | 3.9 | 0.9 |
| 10^-9^ | 48.8 | 7.5 | 7.6 | 2.9 | 5.2 | 1.0 | 4.2 | 1.0 |
| 10^-8^ | 42.0 | 2.6 | 6.4 | 1.1 | 4.8 | 1.1 | 4.1 | 1.2 |
| 10^-7^ | 35.9 | 5.2 | 8.2 | 2.5 | 4.9 | 1.1 | 4.5 | 1.7 |
| 10^-6^ | 29.7 | 3.6 | 9.0 | 3.2 | 5.0 | 1.6 | 4.9 | 1.4 |
| 10^-5^ | 15.6 | 2.4 | 7.8 | 3.4 | 4.9 | 0.8 | 4.1 | 0.9 |
| GA_3_(M) |  | | | | | | | |
| 0 | 33.4 | 9.2 | 6.0 | 1.4 | 5.8 | 1.5 | 4.7 | 1.2 |
| 10^-9^ | 35.1 | 8.1 | 6.3 | 1.2 | 5.5 | 1.4 | 4.8 | 1.2 |
| 10^-8^ | 37.5 | 9.6 | 6.2 | 1.4 | 5.6 | 1.7 | 5.0 | 1.2 |
| 10^-7^ | 35.5 | 3.1 | 6.8 | 1.9 | 5.1 | 1.3 | 5.0 | 1.3 |
| 10^-6^ | 32.8 | 13.1 | 6.8 | 1.7 | 5.3 | 1.6 | 4.6 | 1.2 |
| 10^-5^ | 43.3 | 10.8 | 8.0 | 2.6 | 6.5 | 2.4 | 5.1 | 0.9 |
|  | | | | | | | | |

**Table S3** List of PCR primers used in the present study.

| Gene Name | Squence(5'to3') | Gene Name | Squence(5'to3') |
| --- | --- | --- | --- |
| *NtActin2* | GGATTTGCTGGTGATGATGCT  ATCTTTCTGTCCCATTCCAACC | *Nt*  *CYCA1;1* | TGAGGTTGCCGAGGAATACA  GTCGTTGCCTGTCCATCAAA |
| *Nt*  *CYCD3;1* | ATGAGAGTCCAATAAATAGCCCAAG  ACGAGGTGAATAAGTGGATGGAA | *NtE2Fa* | TCAGCCATGCCTTCAACATC  CCATCAACTCCTCCATTCTCCT |
| *NtH4* | TAGAAGAGGCGGAGTGAAGAGAA  GGTCACAGCATCACGAATCAC | *NtRBR* | GCGGCAAGTGGATAGCAGAA  ACTACAAGCGAGCGTACCAAGAAC |
| *Nt* *CYCB1;1* | TTATGCCTGAGAGCCTTTACCTTAC  GGTGCCCAAATCTCTTCATACTT | *At*  *CYCD3;1* | TCGGAAGGAGGAAGAAAGTAGAGA  TGGAGAAGAAGAAGAAGACAAGGAA |
| *At*  *CYCD3;2* | TTGTTCCTCTTTCTCCTTTCTTCAC ACGCTTCTTCCTCTTTCTCCAA | *At*  *CDKB1;1* | CTAAGTGGGAGCCGCAAGA  AAAGAGGGAAGCAGAAGCAAGA |
| *At*  *CDKB2;1* | TGAGAAAGTTGGTGAAGGGACA  CGGAGAGTGGTGGAAGGAAC | *AtE2Fa* | CAAAACCTCCGTTTCATCCTTC  TCTAACAACGACAGCATCTTCCTC |
| *AtDNA polymeras* | GGAAGGAAGCGATGGGAAA  TCCGTCAGCATCCAAATCAA | *AtCDC6a* | CAGAGTGTCAAGTGGTCAAGATGG  TGATTTGTTGGTGTTGAGGTAGAGA |
| *At CDKA;1* | TCATCTCAGCAAATATGCACCC  AGAGCATTCGGTATCCCTGTCA | *AtPP2A* | CATACCCAAGTGCCCCAGA GTAGCAGCAAACTTCCCCAAA |


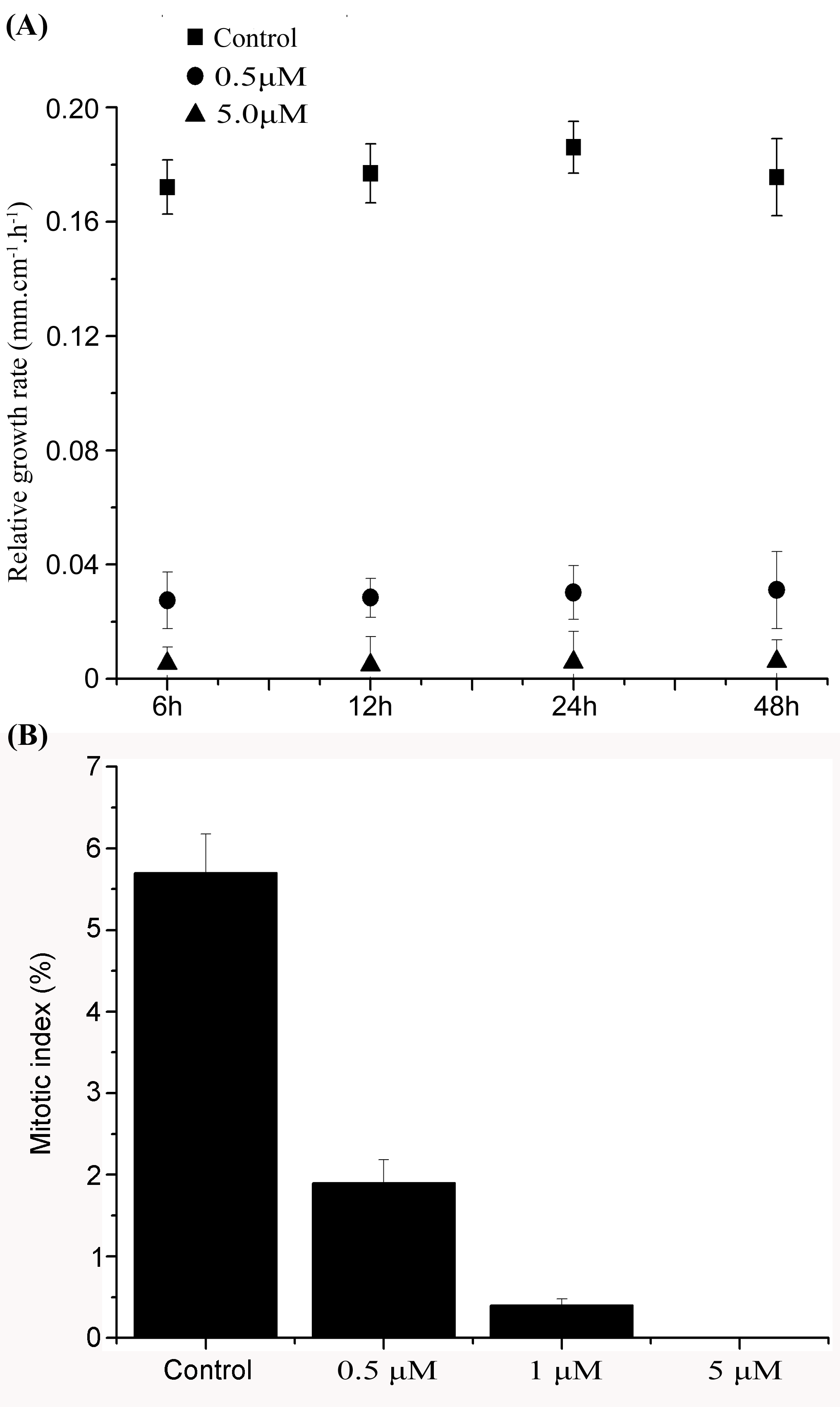


**Figure S1** Effects of NCS on the relative growth rate and mitotic index of *Arabidopsis* roots.

(A) Relative growth rate of *Arabidopsis* roots under different concentrations of NCS. (B) Mitotic index in tips of primary roots of *Arabidopsis*. Results are presented as means ± SE from three experiments. Mitotic index was measured as described by Ding *et al*. [1]. All slides were examined with a Nikon optical microscope.

**
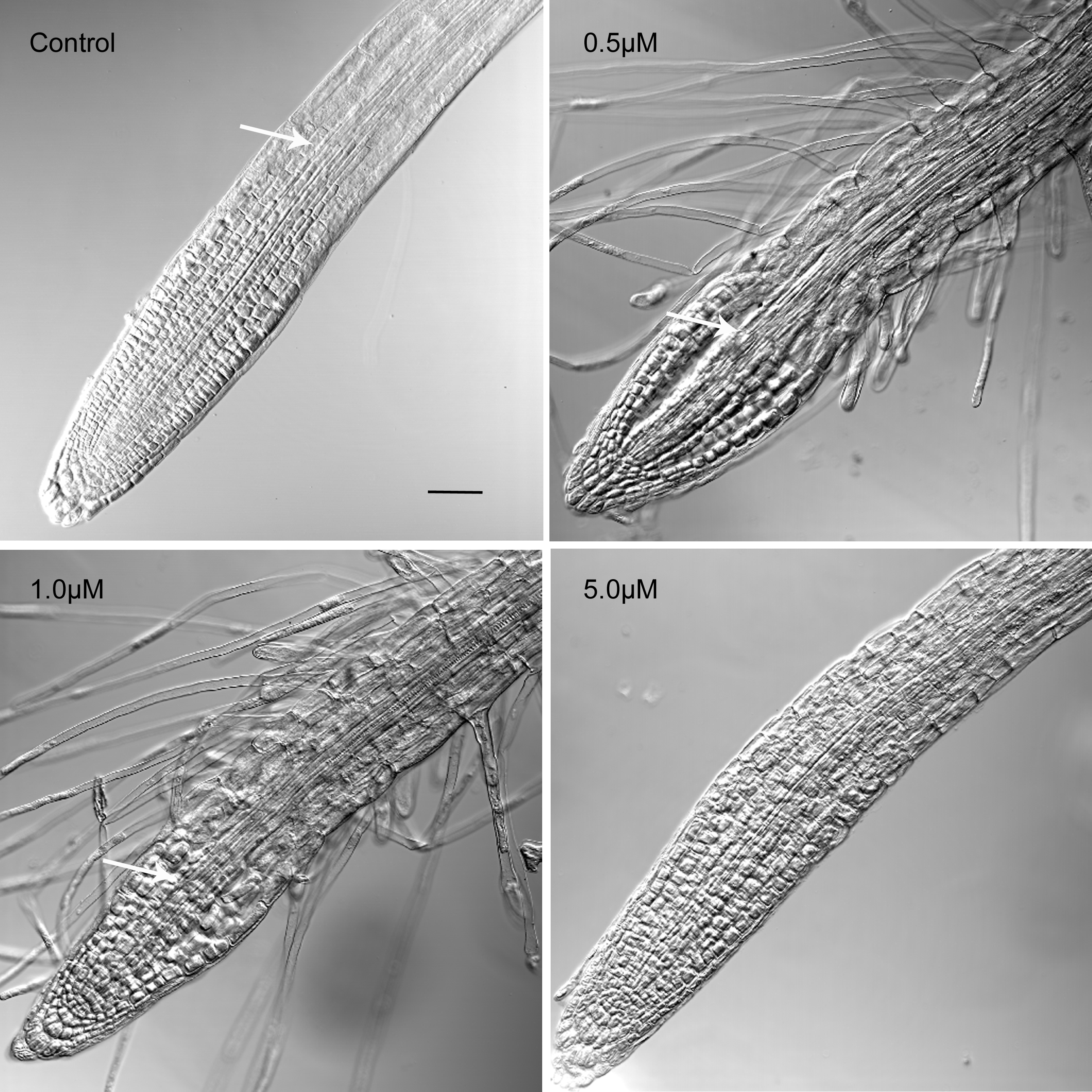
**

**Figure S2** Effects of NCS on differentiation of vascular cells in Arabidopsis roots. Arrows showed the differentiation site of vascular cells. Bar=50μm in all panels. Differential interference contrast (DIC) images were captured using the transmission light detector of the confocal microscope.


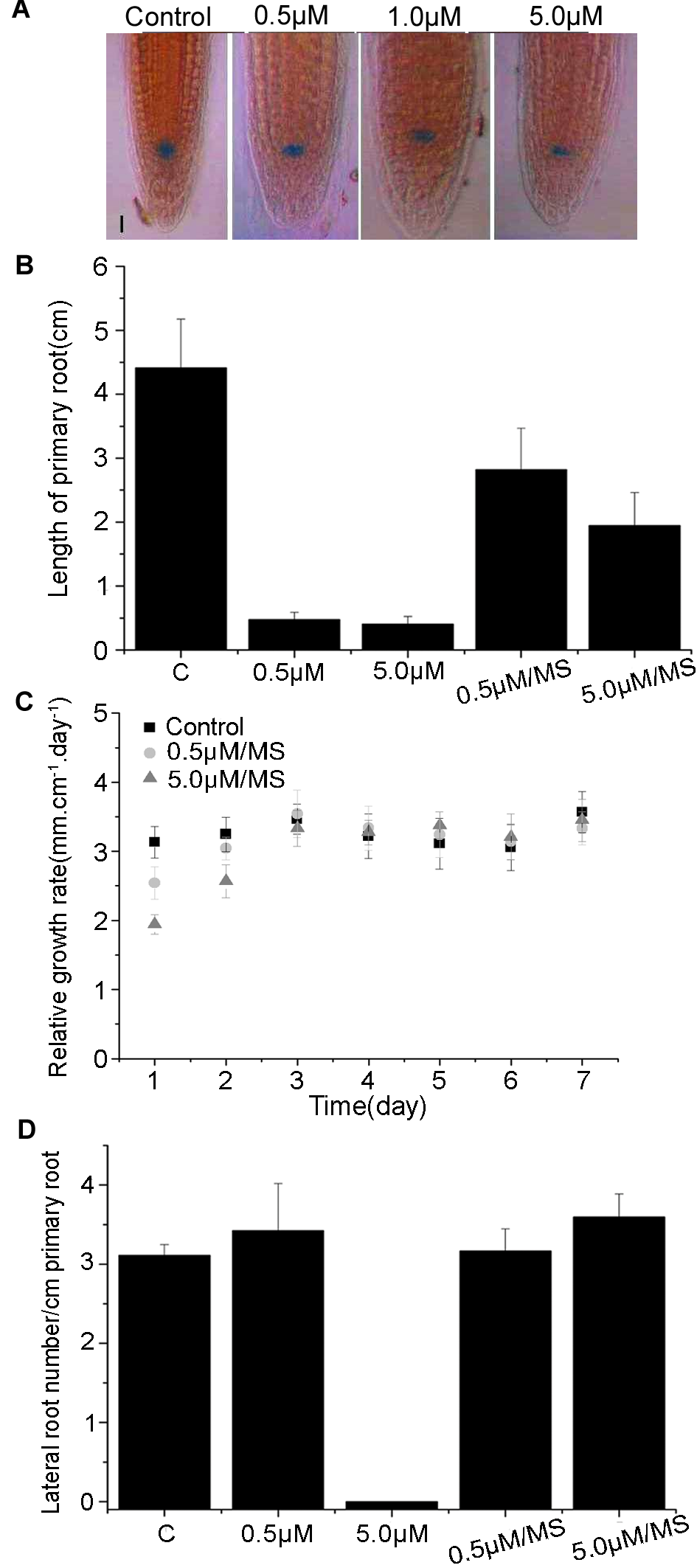


**Figure S3** Recovery of the inhibition effects of NCS on root development. (A) Expression of the *QC* marker, *QC25::uidA*, after NCS treatment. (B) Root length of the NCS-treated *Arabidopsis* seedlings (treated for 4 days) after transferred to 1/2MS medium for 7 days. Data shown are means ± SD of three independent experiments. (C) Relative growth rate of the NCS-treated *Arabidopsis* roots after transfer to 1/2MS medium. Shown are means ± SD of three independent experiments. (D) Lateral root density of the *Arabidopsis* on NCS medium or after transfer to control medium. Data shown are means ± SD of three independent experiments.


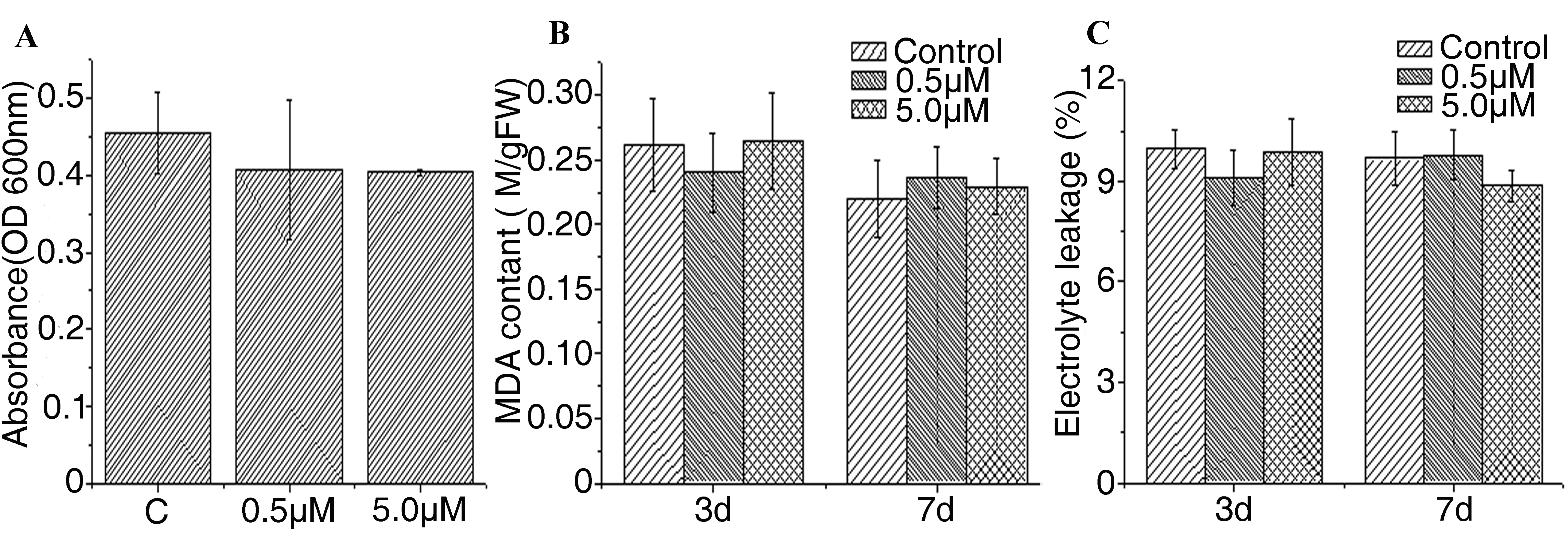


**Figure S4** Physiological effects of NCS in *Arabidopsis* roots.

(A) Cell death rate of *Arabidopsis* roots after NCS treatment for 7 days. Evans Blue-bound dead cells was quantified by A600. Data shown are means ± SD of three independent experiments. (B) Level of lipoxidation product of malondialdehyde (MDA) and (C) electrolyte leakage in *Arabidopsis* roots. Shown are means ± SD of three independent experiments.

Method for detecting the mitotic index

Primary roots of *Arabidopsis* seedlings after NCS treatments were fixed in freshly prepared 3:1 (v/v) ethanol-acetic acid for 24 h, transferred into 70% EtOH, and stored at 4°C until use. Roots were hydrolyzed in 1 M HCl for 8–10 min at 60°C followed by rinsing in distilled water (3–4 rinses). Root tips (approximately 1–2 mm) were excised, stained with carbol fuchsin (10 min), and squashed onto slides. Five slides were prepared for each treatment, and at least 1,000 cells in the meristematic zone were randomly counted per slide. The mitotic index was calculated as the number of dividing cells per 1,000 observed cells.

Reference

1. Ding L, Qi LL, Jing HW, Li J,Wang W, Wang T: **Phytotoxic Effects of Leukamenin E (an ent-kaurene diterpenoid) on Root Growth and Root Hair Development in Lactuca sativa L. seedlings**. *J Chem Ecol* 2008, **34**:1492-1500.
